# Preservice Biology Teachers’ Socioscientific Argumentation: Analyzing Structural and Content Complexity in the Context of a Mandatory COVID-19 Vaccination

**DOI:** 10.1007/s10763-023-10364-z

**Published:** 2023-03-15

**Authors:** Moritz Krell, Carola Garrecht, Nina Minkley

**Affiliations:** 1 IPN – Leibniz Institute for Science and Mathematics Education, Kiel, Germany; 2 Behavioral Biology and Biology Education, Faculty of Biology and Biotechnology, Ruhr-Universität Bochum, Bochum, Germany

**Keywords:** Content complexity, COVID-19 vaccination, Preservice biology teachers, Socioscientific argumentation, Structural complexity

## Abstract

The present study analyzed the structural and the content complexity of 76 preservice science teachers’ socioscientific argumentation in the context of a mandatory COVID-19 vaccination. Data were analyzed within the methodological frame of qualitative content analysis. Concerning the structural complexity, the participants’ socioscientific argumentation reached a relatively high level (i.e., justifications with elaborated grounds). Concerning the complexity of content, the sample referred to science-, ethics-, society-, and politics-related arguments (i.e., almost the full range of content areas); however, on an individual level, participants referred to merely an average of two content areas. Regarding the relationship between structural and content complexity, a significant positive correlation was found. In sum, the results of this study suggest that preservice science teachers’ socioscientific argumentation is on a promisingly high level in terms of structural and on a medium level regarding content complexity. The findings are discussed and implications for science teacher education and assessment in science education are proposed.

**Supplementary Information:**

The online version contains supplementary material available at 10.1007/s10763-023-10364-z.

## Introduction

A major goal of science education is to enable individuals to understand and address science-related issues that are relevant to their personal lives and/or to the broader society in general (e.g., climate change; also known as socioscientific issues, Fleming, 1986; Sadler, 2004). Located at the interface between science and society, socioscientific issues (SSIs) are usually controversial, and their negotiation is often dominated by multiple interest groups (Sadler et al., 2007; Sadler & Zeidler, 2005). To account for the variety of perspectives—and hence the complexity of SSI—the arguments put forward relate to different content areas (i.e., arguments that stem from, e.g., a scientific or an ethical perspective) and mirror different or even contradicting positions (i.e., arguments for or against the issue) (see Christenson & Walan, 2022). Preparing students to actively take part in the negotiation of SSI (i.e., socioscientific argumentation; SSA) has been considered as a central facet of scientific literacy (see Roberts & Bybee, 2014).

In order to plan and conduct lessons that aim to foster students’ SSA, science teachers require the knowledge and skills related to the teaching and the elaboration of these issues (e.g., Kutluca, 2021; Leubecher et al., 2020). Consequently, several studies investigated how science teachers address SSI in their classrooms. Most of these studies suggested that science teachers tend to use SSI as contexts to predominantly foster students’ science content knowledge (e.g., Tidemand & Nielsen, 2017), instead of using them as an opportunity to promote students’ argumentation. The findings of Kara (2012) indicated that this tendency is already evident early in a teacher’s professional career (in undergraduate preservice teacher education). However, focusing merely on the scientific content of SSI does not do justice to their controversial nature and complexity (Kolstø, 2006). Moreover, Han-Tosunoglu and Ozer (2022) emphasized that most research on the elaboration of SSI has been conducted with students. Hence, further studies are needed that investigate (future) science teachers’ knowledge about elaborating socioscientific issues, as they are a key element in promoting SSA in students (Topçu et al., 2010). With the present study, we aim to narrow this gap and used the current situation of the COVID-19 pandemic to examine preservice science teachers' SSA based on the question: Should there be a mandatory COVID-19 vaccination?

There are already some studies in science education on the COVID-19 pandemic as an SSI. For example, Herman et al. (2022) investigated how university biology students’ perceptions about COVID-19 science and sociocultural membership associate with their support for future societal COVID-19 responses. Regarding the individual willingness to get vaccinated, studies have already investigated the influence of factors such as personal risk perception (e.g., Betsch et al., 2019). Unlike these preceding studies, the present study does not mainly aim to provide a deeper insight into preservice science teachers’ thinking and arguments about COVID-19 vaccination. Instead, we are using it as the context to examine the structure and content complexity of preservice science teachers’ SSA.

## Relevance of Socioscientific Argumentation in Science Education

Enabling students to engage with SSI has been described as a central aim of contemporary science education (KMK, 2005, 2020; NGSS Lead States, 2013; OECD, 2019; Zeidler, 2014). To achieve this aim, science education must go beyond the mere provision of the theories, content, and practices necessary for scientific inquiry and must instead give students the opportunity to apply their scientific understanding to real-world contexts (Roberts & Bybee, 2014; Sadler & Zeidler, 2009). From this perspective, students are seen as fully fledged members of society; this further highlights the importance of supporting them “not purely at a knowledge level, but in making decisions and acting as a responsible person” (Holbrook & Rannikmae, 2009, p. 281). Furthermore, negotiating SSI is considered to be cognitively challenging, as SSI characteristically lack straightforward solutions (Kolstø, 2006). Due to their ill-structured nature, these issues can be framed in diverse ways, which incorporate multiple viewpoints (Hoffmann, 2016; Sadler et al., 2007). On the one side, these multiple viewpoints are often rooted in various content areas such as science, ethics, or politics (i.e., content complexity). On the other hand, the ill-structured nature of SSI requires the consideration of different positions as part of the elaboration, such as arguments for or against a certain position (i.e., structural complexity). As documented in many studies, it is this (structural and content) complexity of SSI that causes students to struggle with SSA (e.g., Acar et al., 2010; Evagorou et al., 2012; Nielsen, 2012). Therefore, SSA has become a central focus of science education research (Zeidler, 2014).

One goal of teacher education is to equip teachers with the competencies needed to plan lessons as well as to teach and reflect upon teaching–learning processes professionally (Carlson & Daehler, 2019). These competencies are suggested to encompass professional knowledge (including procedural knowledge and skills), motivational orientations, and self-regulation, as well as beliefs, values, and goals related to teaching and learning (Baumert & Kunter, 2013). With reference to Shulman (1986), professional knowledge can be subdivided into content knowledge (CK), pedagogical content knowledge (PCK), pedagogical knowledge (PK), and further knowledge dimensions (e.g., curricular knowledge). CK is defined as the facts, concepts, and the structure of the subject matter, while PCK refers to knowledge about how to teach a subject (Kind, 2014; Shulman, 1986). As one goal of science education is fostering students’ SSA, science teachers’ professional knowledge goes beyond science knowledge (and how to teach it), but also encompasses relevant knowledge about SSA and about how to teach SSA. Hence, related to SSA, science teachers need to possess relevant CK (e.g., knowledge about ethical values and argumentation), PCK (e.g., knowledge about appropriate teaching strategies and assessment of SSA), and further dispositions, such as beliefs, values, and goals concerning teaching SSA (Albe et al., 2014; Alfs et al., 2012; Gray & Bryce, 2006; van der Zande et al., 2010).

Studies show that science teachers often acknowledge the relevance and importance of SSA for science education (e.g., Bossér et al., 2015; Sadler et al., 2006), but several barriers have been identified, which hinder successful incorporation of SSA in science classrooms. For example, many teachers report limited time for incorporating SSA into the classroom (e.g., Alfs et al., 2012; Ekborg et al., 2013) and a lack of teaching materials (e.g., Alfs et al., 2012; Tidemand & Nielsen, 2017). Furthermore, science teachers reported struggling with teaching SSA because they feel not qualified enough (e.g., Gray & Bryce, 2006; Kilinc et al., 2017) and have only limited ideas about assessing students’ SSA (e.g., Christenson & Walan, 2022; Steffen & Hößle, 2017). Consequently, science teachers tend to use SSI as contexts to foster students’ science content knowledge rather than their SSA (e.g., Ratcliffe & Millar, 2009; Tidemand & Nielsen, 2017). Despite these constraints, several studies report positives cases of incorporating SSA in science classrooms, for example, by adopting communicative (Bossér & Lindahl, 2021) or drama-based (Archila et al., 2022) teaching approaches.

While there are numerous studies on students’ SSA (e.g., Agell et al., 2014; Christenson et al., 2014; Garrecht et al., 2021), much less is known about (preservice) science teachers’ abilities regarding SSA (Han-Tosunoglu & Ozer, 2022). Existing studies predominantly focus on (preservice) science teachers’ PCK (e.g., knowledge about assessment; Steffen & Hößle, 2017) or their beliefs, values, and goals concerning teaching SSA (e.g., importance of SSA for science education; Sadler et al., 2006). Much less is known about (preservice) science teachers’ knowledge and abilities related to SSA (i.e., CK). Studies on teacher education have found that teachers’ CK is a significant predictor of teaching quality and student learning (e.g., Gess-Newsome et al., 2019). Hence, teachers’ abilities to unravel the complexity of SSI within their own SSA might be of great importance in order to enable them to design appropriate learning opportunities for students (see Sadler, 2004).

## Structural and Content Complexity of Socioscientific Argumentation

To analyze the structural complexity of an argument, most researchers apply Toulmin’s argument pattern (TAP; Chinn, 2006; Toulmin, 1958). Based on this framework, individual arguments are examined in terms of different structure components (i.e., data, warrant, backing, rebuttal, and claim). Other studies have used similar frameworks to interpret the structure of arguments (e.g., Baytelman et al., 2020; Capkinoglu et al., 2020), with some of them putting greater emphasis on the dialectic nature of argumentation (e.g., Evagorou & Osborne, 2013; Zohar & Nemet, 2002). In addition to evaluating the structure of an argument based on these interconnected components (i.e., on the micro level), they can also be analyzed by considering the interplay of several arguments, focusing on elements such as the reason(s) for and against a position, an anticipation of the consequences, and a reflection on the argumentation process (i.e., on the macro level; e.g., Reitschert et al., 2007; Sadler & Fowler, 2006). However, it has been criticized that concentrating on the mere “architecture” of an argument often oversees its content (Jafari & Meisert, 2021; Sampson & Clark, 2008). In response to this criticism, frameworks have been established that place emphasis on the content of an argument. One such framework, which has frequently been used in the context of SSA, is the SEE-SEP model, which covers the different content areas of an argumentation process, namely, the social/cultural, environmental, economic, scientific, ethical, and political aspects (Chang Rundgren & Rundgren, 2010).

In the present article, based on existing frameworks, we define structural complexity as the interplay between several arguments that are considered in the argumentation process (e.g., Reitschert et al., 2007; Sadler & Fowler, 2006), increased structural complexity is displayed by a greater interplay of arguments. Furthermore, we define content complexity as the number of different content areas that are part of the argumentation; accordingly, content complexity is higher when a greater number of different content areas are considered.

## Studies That Have Investigated the Structural and the Content Complexity of Preservice Science Teachers’ Socioscientific Argumentation

Only a very limited number of studies so far specifically addressed the structural (e.g., Cetin et al., 2014; Ceyhan et al., 2021; Topçu et al., 2010) and the content (e.g., Ladachart & Ladachart, 2021; Lee et al., 2012; Topçu et al., 2011) complexity of preservice science teachers’ SSA. For example, Topçu et al. (2010) used the TAP (Toulmin, 1958) as a starting point to develop a framework to analyze preservice science teachers’ SSA in seven different scenarios (related to gene therapy, human cloning, and global warming) on the structural level, without considering the content areas in the arguments. The framework included four levels of structural complexity, with the lowest level for arguments that merely include a claim. Arguments that were assigned to higher levels, increased in complexity by also including justifications, counter positions, and rebuttals. The results of Topçu et al.’s (2010) study revealed that the participating preservice teachers most often formulated claims with justifications (level 2).

Other studies investigated the content complexity of preservice science teachers’ argumentation in SSI (e.g., Ladachart & Ladachart, 2021; Lee et al., 2012; Topçu et al., 2011). Although such studies that specifically examine the content complexity are rare, of course all studies that deal with argumentation also deal with content aspects in some way. The SSI used in studies dealing with content complexity range from environmental and climate-related issues such as nuclear power (e.g., Ozturk & Yilmaz-Tüzün, 2017), through human cloning (e.g., Topçu et al., 2010), to abortion (e.g., Betul Cebesoy & Chang Rundgren, 2021). Ladachart and Ladachart (2021), for example, explored preservice biology teachers’ decision-making and informal reasoning on two culture-based SSI, namely, floating vessels into rivers and releasing lanterns into the sky. Their analysis was conducted based on four topics, including personal, cultural, social, and environmental considerations. The participants considered multiple perspectives in both issues and argued for or against an issue depending on the issue. Betul Cebesoy & Chang Rundgren (2021) investigated the content complexity of preservice teachers’ argumentation in three scenarios dealing with abortion. Their analysis with the SEE-SEP model showed that the decisions were mainly influenced by science and ethics/morality, and arguments from the other content areas were only rarely mentioned, independent from the scenario and whether they decide for or against the abortion. However, the extent to which individual participants use arguments from different content areas was not investigated.

Some studies also investigated both the structural and the content complexity of preservice science teachers’ SSA. For example, Cinici (2016) reported findings from a preservice elementary teacher program on SSA in the context of genetically modified organisms (GMOs). Next to other measures, the author analyzed the participants’ argumentation qualitatively, with a focus on the scientific perspective of the arguments (i.e., whether key concepts of science were included) and a structure analysis identifying the number of scientific reasons given for and against GMOs (without considering whether these reasons originate from different content areas). Additionally, the author did not report any relationship between the content and the structure of the preservice teachers’ arguments. In another study, Ozturk and Yilmaz-Tüzün (2017) investigated preservice elementary science teachers’ informal reasoning in an SSI relating to nuclear power usage, along with their epistemological beliefs. They analyzed participants’ argumentation in terms of decision-making modes (i.e., intuitive or evidence-based), reasoning modes (i.e., content of arguments), and reasoning levels (i.e., structure of argumentation). They found that about 90% of the participants showed evidence-based reasoning and they observed six reasoning modes: social-, economic-, ecology-, science- or technology-oriented arguments, types of risk arguments, and political-oriented arguments. Concerning the reasoning levels, the participants, on average, provided about eight arguments, including initial supportive and counterarguments, supportive and counterarguments,, and rebuttals. However, an analysis of the relationship between the different modes and the levels was not conducted.

To summarize, only few studies so far addressed the structural complexity of preservice science teachers’ SSA (or related abilities, such as decision-making). Moreover, most of these studies analyzed the arguments on a micro level (i.e., based on the TAP; Toulmin, 1958) and did not regard the interplay of several arguments on a macro level. The content complexity of preservice science teachers’ SSA has been addressed in some studies with various analytical frameworks; however, to the best of our knowledge, only few studies so far have highlighted which content areas are touched upon on an individual level. Finally, very few studies analyzed both the structural and the content complexity. However, an investigation of the relationship between the structural and the content complexity might be particularly relevant because it could provide a comprehensive picture of preservice science teachers’ argumentation and has the potential to examine the complexity of preservice science teachers’ SSA processes more holistically.

## Aims of This Study and Research Questions

As shown above, the number of studies that have addressed preservice science teachers’ argumentation in SSI regarding structural and content complexity is limited, and there is only sparse evidence on the relationship between their content and structural complexity. However, the relationship between the two dimensions may be of particular interest, since high-quality arguments in the context of SSI need to be appropriately constructed in terms of both structure and content (see Capkinoglu et al., 2020; Christenson & Chang Rundgren, 2015; Christenson et al., 2017). Since previous studies have mostly focused on only one or the other dimension separately, there is a lack not only of a holistic perspective on SSA, but also of insights into the possible interdependence of the two dimensions. Therefore, the present study sets out to explore both aspects and their relationship in the context of a mandatory COVID-19 vaccination in order to (1) analyze the structural and the content complexity of preservice science teachers’ SSA and to (2) examine their relationship. The following research questions (RQ) were addressed in this study:

RQ1: What is the structural complexity (i.e., the level of interplay among arguments) of preservice science teachers’ argumentation in the context of a mandatory COVID-19 vaccination?

RQ2: What is the content complexity (i.e., the diversity of content areas) of preservice science teachers’ argumentation in the context of a mandatory COVID-19 vaccination?

RQ3: What is the relationship between the structural and the content complexity of preservice science teachers’ argumentation in the context of a mandatory COVID-19 vaccination?

## Methods

### Study Context

This study was conducted in the context of teacher education in Germany. In Germany, preservice teachers have to complete a six-semester bachelor’s program, followed by a four-semester master’s program (concurrent teacher education programs). At the end of their studies, they are expected to have developed the basic professional knowledge and competences needed for their profession (Neumann et al., 2017). For example, they are expected to be able to factually and ethically evaluate biological issues in different contexts, and to justify the individual and societal relevance of them (KMK, 2019, p. 22).

### Data Collection and Sample

Preservice biology teachers at a public university in Germany participated in this study. Preservice biology teachers at the respective university learn about the curricular relevance of SSI and SSA as well as appropriate teaching approaches to foster students’ SSA (see Leubecher et al., 2020). However, because of the manifold aims of science education in Germany (KMK, 2005, 2020), these opportunities to learn about SSA provide a rather small glimpse into this area of science education, which needs to be further developed during the preparatory service and the following time as fully qualified teacher at schools.

Preservice teachers were invited to participate in this study via email, using the course lists of one bachelor’s (February 2021, *N* = 173) and one master’s module (May 2021, *N* = 80). Both modules were offered online (but synchronously) and included several seminars.

**Fig. 1:**
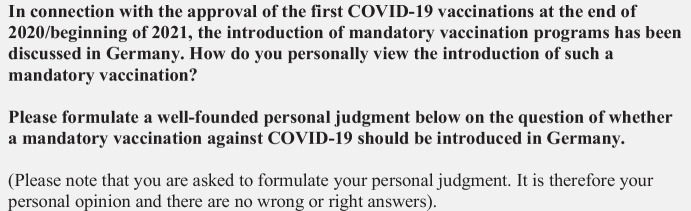
Prompt used for data collection (Please note that the study was conducted in German and that the prompt presented here is a translation of the original prompt.)

The participants were requested to formulate a well-founded personal judgment on the question of whether a mandatory vaccination against COVID-19 should be introduced in Germany (Fig. 1); participation was voluntary and anonymous.

All participants studied biology as one of their subjects (i.e., they were preservice biology teachers). Their second subject was in different fields, with social science/philosophy being the most common (*n* = 31), followed by languages (*n* = 21) and subjects within the STEM disciplines (*n* = 12). The data analysis did not reveal any differences in the findings between participants with different subjects. Therefore, the second subject is not addressed in the following.

A total sample of *N* = 76 preservice biology teachers agreed to participate (*n* = 64 from the bachelor’s program, *n* = 12 from the master’s program). The lower response rate in the master’s group (about 15%) compared to the bachelor’s group (about 37%) is most likely explained by their high work load towards the end of the teacher education program, including practical semester and master’s thesis.

### Discussion About the COVID-19 Vaccination in Germany at the Time of Data Collection

At the end of the two survey periods (February and May 2021), public discussion in Germany was dominated by the lack of vaccines; at this time, only a small number of people had been vaccinated (BMG, 2021). Because there were not yet enough vaccines available for everyone, there was a strict prioritization process, based on the risk assessment of a severe course of COVID-19 (i.e., older people and those with previous illnesses were vaccinated first). Until early March 2021, only people with the highest vaccination priority (e.g., over 80 years old, hospital staff) received a vaccination appointment (STIKO, 2021). As most students do not belong to this group, a large proportion assumed that they would have to wait for their own vaccination. In addition, there was a real run on vaccinations (with people also trying to illegally get an earlier vaccination) so that it was assumed that a large part of the population would get vaccinated voluntarily, which is why a mandatory vaccination did not seem necessary at that time. In addition, at that time, the virus variants against which the vaccination had been developed were most common, there was hardly any medication for COVID-19, and it was assumed that a herd immunity could be achieved if enough people were vaccinated, so that even those who cannot get vaccinated (e.g., because of certain pre-existing conditions) would be protected. At the same time, there was a certain skepticism towards the first developed and approved vaccines (m-RNA and vector), which resulted from the fact that both methods are quite new, have never been used as human vaccinations before, and the vaccine development and approval process was much shorter than usual (Frankfurter Allgemeine, 2021).

More generally, the debate about a mandatory vaccination can be a complex and cognitively challenging issue (German Ethics Council, 2020, 2021). On the one hand, discussing whether a vaccination (e.g., in the context of COVID-19) should be mandatory requires the deliberation of arguments that refer to different disciplines, such as scientific (e.g., herd immunity), ethical (e.g., freedom of choice), and social (e.g., division of society) disciplines. On the other hand, several and sometimes opposing perspectives need to be considered as there are valid arguments both in favor of and against a mandatory vaccination.

### Data Analysis

In the present study, we categorized preservice biology teachers’ argumentation according to its structural and content complexity. Therefore, we build upon two existing and established category systems that have been proposed for the analysis of students’ argumentation in SSI and adapted them to our context. Concerning structural complexity, we used the scheme proposed by Sadler and Fowler (2006); concerning content complexity, we used the SEE-SEP model (Chang Rundgren & Rundgren, 2010). In a third step, we statistically analyzed the data using nonparametric statistics as the data are not normally distributed (indicated by the Shapiro–Wilk test). Spearman correlation analyses were conducted to examine the relationship between the structural and the content complexity and the Mann–Whitney *U* test was used to explore differences between preservice biology teachers from the bachelor’s and from the master’s program.

### Structural complexity

For the analysis of the structural complexity, the coding scheme by Sadler and Fowler (2006) was applied. There are five levels of structural complexity (0–4) (Table 1; see Electronic Supplemental Material 1 for the full coding scheme). Level 0 is defined as having no position at all. In contrast, on level 1, one’s position/opinion is further elaborated upon, but not clarified or justified. Hence, a justification for the position/opinion is missing. On subsequent levels, the position/opinion is clarified or justified, but not thoroughly and only regarding at least one specific aspect of the topic (level 2), it is clarified or justified thoroughly (level 3), or, on the highest level 4, it is clarified or justified thoroughly and also considers arguments of the counter position.

**Table 1:** Level descriptions for the coding scheme by Sadler and Fowler (2006) to analyze structural complexity (see Electronic Supplemental Material 1 for the full coding scheme)

Level	
0	No justification
1	Justification with no grounds
2	Justification with simple grounds
3	Justification with elaborated grounds
4	Justification with elaborated grounds and a counter position

Hence, the levels differ regarding the structural complexity of the argumentation. It starts with the absence of a justification for one’s position and, as the level increases, the complexity of the argumentation increases (e.g., by giving examples, giving detailed reasons, etc.). For reaching the highest level, it is required to consider the opposing position in addition to detailed reasoning.

Our data analysis was conducted following the approach of qualitative content analysis and considering different quality ensuring procedures (Göhner & Krell, 2020; Schreier, 2012). First, a well-established coding scheme was used. Second, independent coding was done by two trained student assistants and cases of disagreement were used to discuss and adapt the coding scheme, that is, to sharpen category descriptions for the present research context and to add sample statements. Third, another round of coding was done by the third author using the optimized coding scheme. Comparing this coding with the student assistants’ consensus, the Kappa was calculated as a measure of intercoder agreement (Brennan & Prediger, 1981), indicating “moderate” intercoder agreement (K = 0.44; Landis & Koch, 1977). Finally, all cases of disagreement were resolved by discussion to achieve a consensus.

### Content Complexity

For the analysis of the content complexity, we applied the SEE-SEP model of Chang Rundgren and Rundgren (2010), which is designed to investigate the elaboration of SSI. According to Chang Rundgren and Rundgren (2010), coming to an informed view on SSI should include recognizing the various problem-dependent perspectives, which are often rooted in different content areas. To examine preservice biology teachers’ multidisciplinary investigation of a mandatory COVID-19 vaccination, the decisions were evaluated in terms of the following six content areas: sociology/culture (So), environment (En), economy (Ec), science (Sc), ethics/morality (Et), and policy (Po) (Chang Rundgren & Rundgren, 2010). For example, in this context arguments could build on constraints on public life (sociology/culture; So) or on the expected economic impact of the COVID-19 crisis (economy, Ec). Sample statements for each of the content areas can be found in Electronic Supplemental Material 2.

Building upon the SEE-SEP model and relevant literature (e.g., Christenson et al., 2014), we first developed a coding scheme for the issue of a mandatory COVID-19 vaccination. This coding scheme included coding rules as well as sample statements for each of the content areas. Thereafter, the coding scheme was discussed and revised several times with a student assistant before it was used to analyze each of the argumentation with regard to (1) the content area (i.e., reference to one of the six content areas) and (2) whether they argued for or against a mandatory vaccination. Next, intercoder agreement was calculated. For this purpose, a second rater (another student assistant) was trained on how to use the coding scheme before coding about 25% of the material. The intercoder agreement was found to be “fair” for the category of ethics/morality (K = 0.40) and “moderate” to “almost perfect” for the other categories (0.63 ≤ K ≤ 1.0; Landis & Koch, 1977). Deviating results were re-examined by one of the authors and both student assistants until a common agreement was found.

To analyze the content complexity, we followed a two-step procedure focusing on (1) the total number of arguments that referred to one or more of the six content areas, to get an insight into the content areas frequently used and (2) the diversity of content areas within preservice teachers’ argumentation, to examine the complexity (i.e., the number of different content areas). Specifically, this means that whenever a referral to one of the six content areas was found, that content area was coded as “given” (numerical coding: 1). Conversely, content areas that were not displayed in the argumentation were coded as “not given” (numerical coding: 0). In (the very few) cases where incorrect arguments (e.g., in terms of scientifically correctness) were mentioned, these were also assigned to the respective content area. For each participant, the content areas addressed were summarized to obtain a final score, with a minimum of zero and a maximum of six. When preservice biology teachers referred to strategic ideas on how to improve the current situation, these digressions were not scored because they did not contribute to their decision on whether a mandatory vaccination against COVID-19 should be introduced in Germany. In accordance with our definition, the more content areas were touched upon in the argumentation, the higher was the argumentation’s content complexity.

## Findings

### Structural Complexity

In terms of structural complexity, the participating preservice biology teachers reached relatively high levels: almost 29% (*n* = 22) of the participants reached the highest level 4 and the average level was 2.9 (Table 2).

**Table 2:** Descriptive statistics for structural complexity (L = level; *M* = mean score; *SD* = standard deviation)

	*N*_L0_	*N*_L1_	*N*_L2_	*N*_L3_	*N*_L4_	*M*	*SD*
Level of structural complexity	1	4	18	28	22	2.90	0.95

In the following, sample responses are provided for each level:

- Level 0: *With vaccinations, you need to proceed with caution*. (An opinion is expressed without providing any further elaboration.)
- Level 1: *I think there should not be a mandatory vaccination but everybody should make a commitment to get vaccinated as long as there is no valid reason not to get vaccinated. A valid reason can be that one simply does not want it. Otherwise, vaccination should be standard as with other vaccinations*. (The position is elaborated upon in more detail but no justifications are given.)
- Level 2: *I personally feel that vaccination is a milestone in medical development and I approve of it. However, I find it controversial to introduce a mandatory vaccination. It would be an encroachment on individual freedom. Every person should have the opportunity to decide themselves which substances are injected into their body. Of course, one can also be of a different opinion, because there are already mandatory vaccinations, such as the measles vaccination. Ultimately, however, I think the decision should not be made by the state and vaccination should not be mandatory.* (The position is justified, but not thoroughly and only regarding the aspect of individual freedom. The aspect of individual freedom is comprehensively elaborated upon.)
- Level 3: *Individual freedom is a great asset in our democracy and society. However, the structure in which we live can only function if solidarity and togetherness are fundamental elements in it. I think that interventions in the body based on coercion represent the greatest curtailment of freedom and are morally questionable. But I also personally think that the protection of an entire population takes precedence over individual freedom. With the COVID-19 restrictions, for example, the economic and psychological consequences for individuals were also not given as much importance as the protection of life in general. Therefore, I am in favor of a mandatory vaccination for the protection of all and especially of those who cannot get vaccinated*. (The position is clarified more thoroughly by pointing out several aspects of the topic, such as individual freedom and protection of the entire population, and giving reasons for the decision, including a profound elaboration.)
- Level 4: *The first question is: How can we ensure a high willingness to get vaccinated, or how can we ensure that a sufficient proportion of the population is vaccinated so that herd immunity against COVID-19 is achieved? At first glance, it may seem logical and simple to introduce a mandatory vaccination in order to achieve a really high vaccination rate. However, on closer examination, this would have some negative consequences. On the one hand, many people would feel deprived of their freedom of choice and their right to physical integrity. The already comparatively high level of vaccination skepticism in Germany would continue to rise in response to a mandatory vaccination and the increasing number of conspiracy theorists would probably continue its trend. A mandatory vaccination would therefore not be readily accepted by the population and could lead to protests and, on the one hand, to a great deal of skepticism toward decisive politicians and, on the other hand, to general skepticism about vaccinations. This could, in turn, have a negative effect on the willingness to get vaccinated against important diseases, especially against childhood diseases. I therefore think it makes much more sense to educate people and raise public awareness so that they can overcome their skepticism. It has become apparent here, and not just since COVID-19, that this work should be done more effectively. On the one hand, the population should be able to understand exactly how vaccination works and why a vaccination is much less harmful than the disease itself; on the other hand, it is important that people understand the concept of herd immunity. In my opinion, this educational work should not only take place *via* the media but should also find its way into the education system. In summary, therefore, I would argue against a mandatory vaccination but, at the same time, would argue for a focus on more education and “persuasion” work*. (The position is clarified thoroughly and also contains arguments of the counter position, such as reaching a high vaccination rate and, hence, herd immunity.)

There was a significant difference in the level of structural complexity between preservice teachers in the bachelor’s (*M* = 2.80; *SD* = 0.98) and those in the master’s program (*M* = 3.42; *SD* = 0.52); *U* = 235; *z* =  − 2.05; *p* = 0.040; *d* = 0.50, medium effect size.

### Content Complexity

In total, 200 arguments were coded to one of the six content areas. On average, preservice biology teachers provided about 2.5 arguments.

From all arguments, nearly two thirds (*n* = 119) were coded as being against a mandatory vaccination, whereas *n* = 81 supported the position for a mandatory vaccination.

**Fig. 2:**
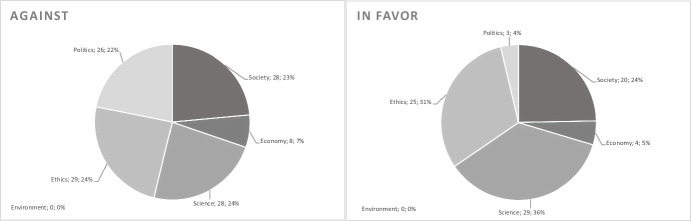
Distribution of six content areas mentioned in the argumentation against (left) and in favor of a mandatory vaccination (right). Note that environment-related arguments were not found in this study

The disciplines that were referred to most often were science (Sc; *n* = 57), ethics (Et; *n* = 54), and society (So; *n* = 48). The area of politics accounted for a quarter of the arguments against but only for 4% of the arguments in favor of a mandatory vaccination. The other areas were mentioned by both those in favor and those against. In the arguments against a mandatory vaccination, the areas of science, society, and ethics were distributed equally (each about 24%), while there was a certain degree of dispersion in the arguments in favor. Here, the arguments from the science area (36%) outweighed the ones from the ethical (31%) and social (25%) areas (Fig. 2).

Regarding the content complexity, we concentrated on the number of different content areas that the preservice biology teachers referred to in their SSA. On average, they touched upon 2.24 content areas (*SD* = 1.03). No significant difference in the number of different content areas was found between preservice teachers in the bachelor’s (*M* = 2.14; *SD* = 1.01) and those in the master’s program (*M* = 2.75; *SD* = 1.06); *U* = 267.50; *z* =  − 1.73; *p* = 0.084; *d* = 0.39, small effect size. Within our sample, the maximum of references to different content areas was four (Table 3).

**Table 3:** Frequency of references to different content areas

Number of content areas per decision	*N*
0 content areas	2
1 content area	17
2 content areas	28
3 content areas	19
4 content areas	10

In the following, sample responses are provided for each content area:

- Society-related: *I think one rather reaches [with a mandatory vaccination] a negative mood among citizens and there is more place for conspiracy theories.*
- Economy-related: *[Mandatory vaccination] would relieve the economy, the educational system and above all the individuals who have financial and family problems.*
- Science -related:* I am against mandatory vaccination, especially for a vaccine that has only recently been tested in practice and could potentially carry risks.*
- Ethics-related: *[…] because then I also protect very many other people who could be infected by me.*
- Politics-related: *On the one hand, mandatory vaccination is an encroachment on the right of freedom of a person.*

### Relationship Between Structural and Content Complexity

**Fig. 3:**
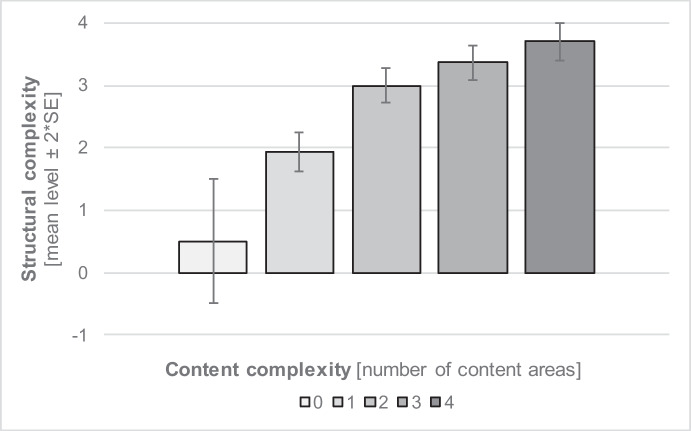
Mean structural complexity in each level of content complexity

The joint evaluation of structural and content complexity revealed significant positive correlations between the structural complexity and the number of content areas per argumentation (*r* = 0.67, *p* < 0.001; large effect size). Figure 3 illustrates the rather linear relationship between both measures. The rather high variance (two times the standard error) of the reached level of structural complexity for those participants who mentioned zero content areas is due to the fact that there are only two such participants (see Table 3).

## Discussion

This study analyzed the structural and content complexity and their relationship of preservice science teachers’ SSA in the context of a mandatory COVID-19 vaccination. As shown in the review of the literature, there is only limited evidence on preservice science teachers’ argumentation in SSI and only a few studies analyzed both the structural and the content complexity of their argumentation thus far. However, knowledge about this relationship might be of high relevance because it can provide a more holistic picture of preservice science teachers’ argumentation in SSI. To address this gap, preservice biology teachers were asked to formulate a reasoned personal judgment on the question of whether a mandatory vaccination against COVID-19 should be introduced in Germany.

In terms of the structural complexity (RQ1), our findings indicate that the preservice science teachers’ SSA was on a relatively high level. Most of the participants provided justifications with elaborated grounds and many of them also considered opposing shifts in perspectives by incorporating reasons for and against a mandatory vaccination or their own position, respectively. These findings are encouraging as they are indicating a slightly higher level of structural complexity in preservice science teachers’ SSA than reported in previous studies, where participants scored predominantly on the medium levels (e.g., Ceyhan et al., 2021; Topçu et al., 2010). We also found that the preservice science teachers from the master’s program reached a significantly higher level of structural complexity than those in the bachelor’s program. This might indicate that the opportunities to learn about argumentation during the teacher education program have a positive effect on the preservice teachers’ ability to argue in a more complex way. However, as master students are typically older than bachelor students this finding might also indicating a developmental effect. Moreover, the group of master students was smaller than the group of bachelor students, which may have led to a statistical bias.

In terms of the content complexity (RQ2), the findings can be viewed regarding the reference to content areas on a group level (i.e., distribution of arguments among the six content areas) and on an individual level (i.e., frequency of references to the six content areas). Across all content areas, science-, ethics-, and society-related arguments were frequently used in both arguments for and against a mandatory vaccination. The frequent use of science-related arguments is especially promising, as previous studies on students’ SSA have shown that they have difficulties to propose science- and technology-oriented arguments in SSI contexts (e.g., Lewis & Leach, 2006; Wu & Tsai, 2007). Wu and Tsai (2007), for example, suggested that students were unable to connect the scientific knowledge they had learned in school with the discussion of authentic SSI. Our findings, in contrast, provide evidence that preservice teachers often used scientific arguments in the context of a mandatory vaccination, which may suggest that they are prepared to use this SSI to explore the connection between science and society with their future students. Looking particularly at the arguments against a mandatory vaccination, the participants used arguments that were almost evenly related to four of the six content areas assessed. Considering the fact that arguments that from the environment content area can only be generated with some difficulty in the SSI we used for our investigation, the group of preservice biology teachers touched upon almost all areas (4 out of 5) as part of their argumentation. This finding seems interesting given that, in a previous study, findings provided evidence that students—even after an explicit intervention to improve their familiarity with a particular SSI—preferred certain content areas and that, in order to broaden their use of diverse content areas, more instructional guidance appeared to be necessary (Garrecht et al., 2021). However, although our sample referred to most of the content areas on the group level, it appears that most preservice teachers addressed only two different content areas on the individual level (Table 3). A quarter of the participants (*n* = 19) only referred to one or none content area. This finding indicates that it seems necessary to develop suitable measures that assist preservice teachers to increase their content complexity in SSA. No difference in the number of mentioned content areas was found between participants from the bachelor’s and the master’s program.

Concerning the relationship between the structural and content complexity (RQ3), a significant positive correlation was found. This indicates that the consideration of various content areas was associated with a more structurally complex argumentation, and vice versa. As correlation cannot explain causation, the specific mechanism of this relationship has to be investigated in future studies. One possible explanation is that, often—and also in the case of a mandatory COVID-19 vaccination—arguments for and against a position come from different content areas and, hence, including these arguments results in higher content complexity (i.e., high structural complexity causes high content complexity). However, it could also be the other way around. Students who are arguing from the perspective of different content areas are, therefore, including opposing positions in their argumentation (i.e., high content complexity causes high structural complexity). Independent from the specific mechanism behind the positive correlation between structural and content complexity, our findings suggest that both aspects are strongly associated. Hence, it can be assumed that both aspects can be introduced and discussed jointly in science teacher education courses.

## Limitations and Future Studies

Before discussing the implications of our results, some of the study’s shortcomings should be discussed. Most importantly, only one open-ended question was used in this study, asking the participants to formulate their personal judgment on the question of whether a mandatory vaccination against COVID-19 should be introduced in Germany. Hence, the present findings are not generalizable beyond this context. The timing, the country, the availability of vaccines, and, generally, the phase of the pandemic in which the survey took place play also a major role and need to be carefully considered when interpreting and discussing participants’ SSA in this context. At the time of our survey, for example, effective vaccinations were already available, but there were not yet enough doses for everyone. In addition, it was not yet possible to predict how strong the willingness to get vaccinated would be among the population once vaccines were available for everyone. Finally, as the COVID-19 pandemic and the newly developed vaccines were highly prominent in the media, it is likely that the participants were better informed about this SSI than about other SSI that are mentioned less often in the media (e.g., animal testing; Garrecht et al., 2021). It is also likely that individual factors, such as previous illnesses, had an impact on the extent to which participants engage with the issue of vaccination (see Zeidler, 2014). Obtaining this complementary information would have helped to draw a more solid conclusion and is therefore encouraged for further investigations. Last but not least, using a less personal (or not a health-related) topic (e.g., use of nuclear power) could have also resulted in different insights regarding participants’ argumentation in terms of content and structure complexity. Based on the general lack of studies that focused on preservice teachers’ SSA, future studies will have to continue exploring this crucial ability in other contexts, settings, and times.

Methodically, the participants were invited via email and submitted their responses through an online survey. It was emphasized to formulate a personal judgment and that there are no wrong or right answers; however, individual participants might have searched on the internet or discussed with others. We also acknowledge that, although examining the number of *different* content areas is the most appropriate indicator of complexity that meets our definition, it neglects to consider whether a participant mentioned multiple arguments from the same content area. The number of arguments within the *same* content area (i.e., the depth of content areas) could therefore be considered as another way of defining content complexity.

## Conclusion

This study adds to a growing body of research on how students can be prepared to elaborate science-related issues of social relevance by focusing on preservice teachers’ ability to engage in SSA. Moreover, our study is the first to systematically consider the relationship of two different indicators that can be assessed in this regard: structural and content complexity. The finding that the participating preservice teachers reached a relatively high level of structural complexity is encouraging; however, the majority still did not address the various related content areas in their argumentation. This finding supports the call for more explicit consideration of SSA in science teacher education, especially considering the variety of perspectives and related content areas.

## Supplementary Information

Below is the link to the electronic supplementary material.

Supplementary file1 (PDF 127 KB)

Supplementary file2 (PDF 178 KB)

## Data Availability

Data is available upon request to the first author.
