# Supplementary material for: Preservice Biology Teachers’ Socioscientific Argumentation: Analyzing Structural and Content Complexity in the Context of a Mandatory COVID-19 Vaccination
Source: Int J Sci Math Educ. 2023 Mar 15:1–21. Online ahead of print. doi: 10.1007/s10763-023-10364-z (PMC10014133; doi:10.1007/s10763-023-10364-z)
Supplement: Supplementary file 1 — Supplementary file1 (PDF 127 KB) [file 10763_2023_10364_MOESM1_ESM.pdf]

Appendix 1

Full coding scheme for structural complexity. Note that the study was conducted in German and that the translations provided here have not been professionally validated. The original German coding scheme is available upon request via email from the first author.

Based on: Sadler, T., & Fowler, S. (2006). A threshold model of content knowledge transfer for socioscientific argumentation. *Science Education, 90*, 986–1004.

Level	Description	Coding rules	Sample quote
0	No justification	The position/opinion is not further elaborated upon.	"With vaccinations you need to proceed with caution."
1	Justification with no grounds	The position/opinion is further elaborated upon, but not clarified or justified.[1]	"I think there should not be a mandatory vaccination but everybody should make a commitment to get vaccinated as long as there is no valid reason not to get vaccinated. A valid reason can be that one simply does not want it. Otherwise, vaccination should be standard as with other vaccinations."
2	Justification with simple grounds.	The position/opinion is clarified or justified, but not thoroughly and only regarding at least one specific aspect of the topic. A justification or clarification contains either current examples or concrete reasons, which are given numerously or are comprehensively elaborated upon.	"In my opinion, vaccination should be mandatory. Especially in light of the new mutations, the infection rate is simply far too high. People who do not get vaccinated are a danger to other citizens (especially children, newborns, etc., who are not yet sure whether they can get the vaccination)."
3	Justification with elaborated grounds.	The position/opinion is clarified or justified thoroughly (e.g., through pointing out several aspects of the topic and giving either reasons for the decision or alternative suggestions; a combination of several criteria or a profound elaboration of one of them is given).	"Personally, I am against a mandatory vaccination against Covid-19, even though I would be very much in favor of everyone receiving this vaccination. However, we are currently in a politically difficult situation where many people are becoming increasingly radicalized because they feel restricted in their personal freedom and there is a general distrust of science. This position is not to be supported, but it must also not be ignored, as it can lead to further radicalization. This is precisely the danger that would arise if a mandatory vaccination were introduced. Opponents of vaccination would not get vaccinated even if mandatory vaccination were introduced and they would get away with it by means of medical certificates or the like. It is important to educate people about vaccinations in the media so that many people decide to get vaccinated themselves because they are doing something good, not just for themselves. I think the possibility to make your own decisions is very important at this point. Otherwise, I would not reject a mandatory vaccination."

4	Justification with elaborated grounds and a counter position.	Beyond the criteria of level 3, the position/opinion also contains arguments of the counter position, which are also reflected on. The extent and detailedness of the reflection may preponderate on one of the sides as long as the counterarguments were substantially elaborated upon.	"Mandatory vaccination would be very helpful in protecting everyone in the population from the disease and would achieve herd immunity faster and would control of the virus. Personally, I am not in favor of a mandatory vaccination in the current period. There are not enough vaccine doses, it is still not clear whether these vaccines have serious long-term consequences, and there is still no vaccine for the younger population. What is most serious, however, is the aspect of not being able to obligate anyone to get vaccinated. Everyone should be free to decide what happens to their body and should not be forced to do anything. The population should be better informed so that there is no room for speculation. However, I think it is good that so-called "threatened parts of the population" such as risk groups are vaccinated. The protection of this part of the population is very important and the vaccine offers a possibility to be able to protect oneself. After all, everyone should protect themselves in this situation, not expose themselves to danger, and think about their fellow human beings."

[1]: A justification is defined as stating circumstances, facts, or the like that have led to a position. This position can be expressed directly or can stem indirectly from the circumstances, facts, or the like.
