# Supplementary material for: Preservice Biology Teachers’ Socioscientific Argumentation: Analyzing Structural and Content Complexity in the Context of a Mandatory COVID-19 Vaccination
Source: Int J Sci Math Educ. 2023 Mar 15:1–21. Online ahead of print. doi: 10.1007/s10763-023-10364-z (PMC10014133; doi:10.1007/s10763-023-10364-z)
Supplement: Supplementary file 2 — Supplementary file2 (PDF 178 KB) [file 10763_2023_10364_MOESM2_ESM.pdf]

Appendix 2: Coding scheme for the different content areas
(based upon the SEE-SEP model by Chang Rundgren & Rundgren, 2010)

**Definition**	**Anchor examples**	**Exemplary topics for coding**
Sociology/Culture (*So*)	"If a mandatory vaccination was introduced, this would divide society more deeply and could only be 'repaired' with great difficulty."<br><br>"I suspect that a mandatory vaccination against COVID-19 would cause great protests and stir up discontent in society."	Rule of coding: Societal aspects are a central part of the argument.<br><br>Exemplary topics (non-exhaustive list):<br>- Restrictions on public life or returning to "normal" public life<br>- Societal impact such as division of society by COVID-19 or vaccination<br>- Negative consequences for a part of society (unvaccinated)<br>- Impact on general vaccination opinion and possibly on willingness to vaccinate in general<br>- Strengthening of conspiracy theories and conflicts within society<br>- There are currently either too few or enough people who are willing to get vaccinated (Note: unless there is also the keyword "herd immunity", then *Sc*)<br><br>Special case: Herd immunity<br>Coded as *So* only if related to return to "normal" life<br><br>Special case: Protection of society<br>When protection of the entire population contrasts with freedom of the individual, the focus is on the aspect of protection (*Et*) and not society (*So*).
Economy (*Ec*)	"A mandatory vaccination would quickly relieve the economy, the education system, and, above all, the individuals who have financial and family problems due to the COVID-19 pandemic."<br><br>"First of all, for a mandatory vaccination to be introduced, the precondition that there are enough vaccines for everyone would have to be fulfilled."	Rule of coding: Economical aspects are a central part of the argument.<br><br>Exemplary topics (non-exhaustive list):<br>- The economic situation or expected economic impact of the COVID-19 crisis on individuals or groups of individuals<br>- Reference is made to the (lack of) availability of vaccines
Environment (*En*)	N/A	N/A

Science<br>(*Sc*)	"[I'm against a mandatory vaccination] especially with a vaccination that has only recently been tested in practice and could potentially bring risks with it."<br>"It would be much more helpful if everyone had to be vaccinated. In this way, a herd immunity would be created much faster and more effectively."	Rule of coding: Scientific aspects are a central part of the argument.<br><br>Exemplary topics (non-exhaustive list):<br>- Side effects or long-term consequences are not yet sufficiently researched<br>- Scientific knowledge regarding, for example, spread of viruses (transmission) or the role of mutations becomes apparent within argument<br>- Critical note that a mandatory vaccination could negatively influence vaccination attitudes (vaccination as a scientific act; loss of confidence in science)<br><br>Special case: Protection<br>Own protection against the virus is coded (*Sc*); protection of others is coded (*Et*)<br><br>Special case: Historical references<br>Historical references (e.g., measles) are only coded if other aspects are also addressed (e.g., herd immunity).
Ethics/Morality<br>(*Et*)	"[…] and therefore saving human lives."<br><br>"[…] then I also protect a lot of other people who could be infected by me."	Rule of coding: Ethical aspects with a focus on values are a central part of the argument.<br><br>Exemplary topics (non-exhaustive list):<br>- Protecting and saving others<br>- Vaccination is considered as a deprivation of freedom<br>- Consideration of common good instead of interest of individuals<br><br>Special case: Freedom<br>Value-based: Argument invokes that people feel restricted in their freedom (*Et*)<br>Enshrined in law: Argument invokes the right to, for example, self-determination (*Po*)
Policy<br>(*Po*)	"[…] however, this contradicts the General Equality Act."<br><br>"On the one hand, a mandatory vaccination is an interference with a person's right to freedom."	Rule of coding: Political or legal aspects or systems are a central part of the argument.<br><br>Exemplary topics (non-exhaustive list):<br>- Right to form opinions, referencing its legal anchoring<br>- General right to live one's own life<br>- Human rights<br>- Right to life and physical integrity

Note: General statements about COVID-19 or on how to improve the current situation that did not address the question of a mandatory vaccination were not coded (e.g., "I guess the mental health risk is much higher right now and should be taken more seriously", "the whole policy and measures should be urgently reconsidered", or "more education is needed!").
